# Binocular Goggle Augmented Imaging and Navigation System provides real-time fluorescence image guidance for tumor resection and sentinel lymph node mapping

**DOI:** 10.1038/srep12117

**Published:** 2015-07-16

**Authors:** Suman B. Mondal, Shengkui Gao, Nan Zhu, Gail P. Sudlow, Kexian Liang, Avik Som, Walter J. Akers, Ryan C. Fields, Julie Margenthaler, Rongguang Liang, Viktor Gruev, Samuel Achilefu

**Affiliations:** 1 Department of Radiology, Washington University School of Medicine, St. Louis, MO, USA; 2 Department of Biomedical Engineering, Washington University, St. Louis, MO, USA; 3 Department of Computer Science and Engineering, Washington University, St. Louis, MO, USA; 4 College of Optical Sciences, The University of Arizona, Tuscon, AZ, USA; 5 Department of Surgery, Barnes-Jewish Hospital, and the Alvin J. Siteman Cancer Center, Washington University School of Medicine, St. Louis, MO, USA; 6 Department of Biochemistry & Molecular Biophysics, Washington University School of Medicine, St. Louis, MO, USA

## Abstract

The inability to identify microscopic tumors and assess surgical margins in real-time during oncologic surgery leads to incomplete tumor removal, increases the chances of tumor recurrence, and necessitates costly repeat surgery. To overcome these challenges, we have developed a wearable goggle augmented imaging and navigation system (GAINS) that can provide accurate intraoperative visualization of tumors and sentinel lymph nodes in real-time without disrupting normal surgical workflow. GAINS projects both near-infrared fluorescence from tumors and the natural color images of tissue onto a head-mounted display without latency. Aided by tumor-targeted contrast agents, the system detected tumors in subcutaneous and metastatic mouse models with high accuracy (sensitivity = 100%, specificity = 98% ± 5% standard deviation). Human pilot studies in breast cancer and melanoma patients using a near-infrared dye show that the GAINS detected sentinel lymph nodes with 100% sensitivity. Clinical use of the GAINS to guide tumor resection and sentinel lymph node mapping promises to improve surgical outcomes, reduce rates of repeat surgery, and improve the accuracy of cancer staging.

Surgical resection is the standard of care for many solid tumors such as breast cancer and melanoma, and sentinel lymph node (SLN) mapping is used for cancer staging^1^. Incomplete tumor removal increases the chances of cancer recurrence and necessitates repeat surgery, whereas inaccurate SLN identification may misdiagnose the cancer stage. Despite recent advances in pre-operative imaging methods, surgeons rely on visual inspection, palpation, and tactile evaluation to distinguish cancerous from uninvolved tissue intraoperatively, leading to subjective decision-making and variable outcomes. For example, 14–50%^2^,^3^,^4^ and 20–70%^5^,^6^,^7^ of patients undergoing melanoma *in situ* and breast conserving surgery, respectively, require repeat surgery. Similarly, inaccurate SLN removal often requires additional surgical interventions^8^. Surgery is most effective when performed before the cancer becomes a metastatic disease. Assessment of the SLN for the presence of cancer cells is a standard of care for staging breast cancer and melanoma^1^. Conventionally, patients are injected peritumorally with ^99 m^Tc sulfur-colloid and a visible blue lymphotropic dye. A handheld gamma-counter is used to localize the region of highest radioactivity, and the blue dye can be used to visualize the SLN. However, radioactive SLN tracking exposes patients and health professionals to ionizing radiation, without SLN visualization capability. Although blue dyes can be visualized, SLN tracking by this method is limited to inspection of only superficial lymph nodes and requires a high dose of the dye, which may lead to harmful side-effects^9^,^10^.

The above challenges have spurred interest in developing methods for accurate intraoperative imaging of tumors and SLNs. Conventional modalities such as magnetic resonance imaging, computed tomography, and positron emission tomography can provide exquisite anatomic and functional information^11^,^12^. However, they are not amenable for use in the operating room (OR) due to their large hardware footprint, specialized operator requirement, prohibitive cost, and the use of ionizing radiation. Slow image reconstruction, lack of microscopic imaging capability, and disruptive information display on a remote monitor affects their wide adoption in the OR for real-time image guidance^13^. Intraoperative ultrasonography can be used for tumor detection based primarily on tissue morphology, leading to significant false positive and negative rates^14^,^15^,^16^,^17^. As a contact based method with relatively poor resolution, intraoperative ultrasonography is less useful for identifying tumor boundaries or microscopic tumors during open surgeries. Advanced instruments that mimic global positioning systems have been developed, where pre-operative computed tomography or magnetic resonance images can be projected onto the appropriate anatomical structures. These systems suffer from limitations of the pre-operative imaging method, unsatisfactory registration due to tissue deformation and motion during surgery, and the inability to directly interrogate boundaries of tumors.

Alternatively, optical imaging uses nonionizing radiation and simple imaging setup for real-time readout and detection of microscopic lesions^18^,^19^. In particular, near-infrared (NIR) fluorescence imaging in the 700–900 nm range is attractive because the low absorption by intrinsic photoactive biomolecules minimizes tissue autofluorescence and facilitates thick tissue assessment. The use of nonionizing radiation decreases safety hazards in the OR^18^,^19^,^20^. Additionally, several tumor-targeted optical contrast agents have been developed^21^ including peptide^22^ and nanoparticle-based agents with promising features^23^. These advantages have generated interest in NIR fluorescence image-guided surgery (NIR-FIGS). To date, several NIR-FIGS systems have been developed, and successfully used for intraoperative tumor imaging and SLN mapping (SLNM), including FLARE^24^, Fluobeam^25^, SPY^26^, and Hamamatsu PDE^27^. However, each of these systems have some limitations, including the use of bulky hardware, potentially disruptive information display on a remote monitor, mismatch between the system’s and surgeon’s field of view (FOV), and require support from other workers to control the device.

Adaptation of head-mounted displays (HMDs) for surgery^28^,^29^,^30^,^31^,^32^,^33^ could overcome the issue of disruptive information display. We have previously demonstrated the feasibility of using wearable cameras and HMDs for FIGS^34^,^35^,^36^. Our initial prototypes used a monocular projection eyepiece, which posed perception problems during surgery, or a binocular system, which only captured white light reflectance and fluorescence images sequentially using the same sensor, thereby preventing display of real-time composite color-fluorescence images. Additionally, the issues of camera and user FOV mismatch, bulky design, non-optimized optics, and fast processing to generate real-time color-fluorescence composite images with minimum latency remain unaddressed. The primary goal of this study is to test the hypothesis that simultaneous, sensitive detection of color and NIR fluorescence information, fast image processing and image output via an HMD would allow non-disruptive access to accurately co-registered color-NIR images for real-time image guidance in oncologic surgery. Toward this goal, we have developed a new wearable goggle aided imaging and navigation system (GAINS) and evaluated the accuracy of using the well-characterized system for real-time intraoperative tumor visualization and image-guided tumor resection in small animals, as well as SLNM in human breast cancer and melanoma patients.

## Results

### Development of GAINS

**Figure 1:**
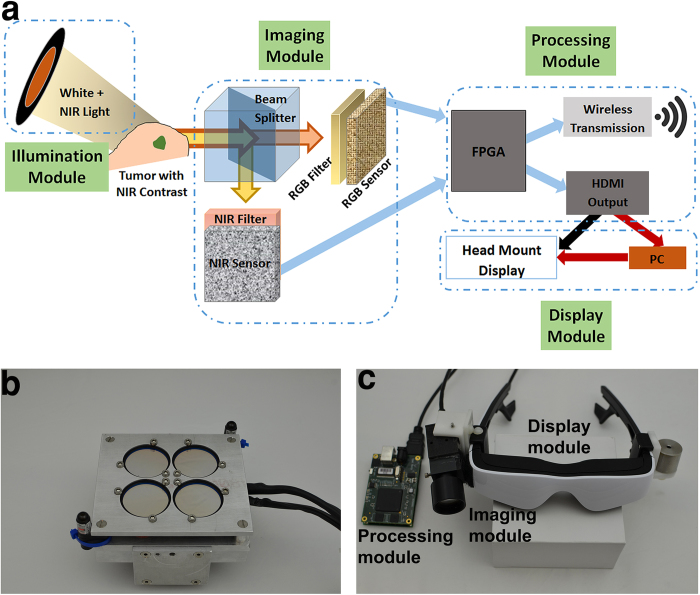
GAINS. (**a**) Schematic demonstrates the information flow through different modules of the system. (**b**) Photograph of the NIR source. (**c**) Photograph of the integrated display and imaging module, along with the processing module, which are worn by the user.

The accuracy of image guidance depends on the sensitivity and resolution of the system, as well as the accuracy of fluorescence to color image overlay. The system detection sensitivity is determined by the fluorescence detection sensitivity because the visible light channel has abundant signal compared to the photon-starved fluorescence channel. The system performance is determined by the sensor and optics of the system, with a smaller sensor pixel pitch and smaller lens aperture leading to higher resolution and larger depth of focus respectively, but with low fluorescence detection. Thus there is a trade-off when using a lower-resolution sensor with larger pixel pitch and large aperture lens with lower depth of focus that allows high fluorescence signal capture for more sensitive detection. Furthermore, the requirement of wearability imposed additional restrictions of having a compact, lightweight, and ergonomic design. These factors precluded the use of different cameras with dedicated lenses for both color and fluorescence channels, and large aperture heavy glass lenses that could capture very large amount of fluorescence signal. The amount of fluorescence signal collected may be increased by using large exposure times. However, the requirement of real-time image guidance constrained the imaging exposure to acquire both fluorescence and color information. Therefore, the challenges in developing the GAINS were achieving sensitive simultaneous imaging in the photon-saturated color channel and photon-starved fluorescence channel, real-time image processing, and non-disruptive display while maintaining a wearable form factor. We overcame these challenges by developing a single lens, color-NIR (Supplementary Fig. S3), and compact lightweight camera (Supplementary Table S1), with independent exposure times for both sensors and a spatial resolution of 320 μm (Supplementary Table S1). GAINS conceptual design is summarized in Fig. 1a. The processing unit generates co-registered composite color-fluorescence images, which are displayed in real-time via a lightweight and high-resolution HMD unit. The NIR source (Fig. 1b) consists of 760 nm light-emitting diodes (LEDs) with 769 ± 41 nm bandpass filter. The display module consists of a 1080p high-resolution HMD. Adjustable mechanical mounting and a counter balance for the imaging module were added to match the camera’s weight and user FOV for improved user experience (Fig. 1c). Additional information on the system development can be found in the Materials and Methods section below.

### *In vitro* studies

**Figure 2:**
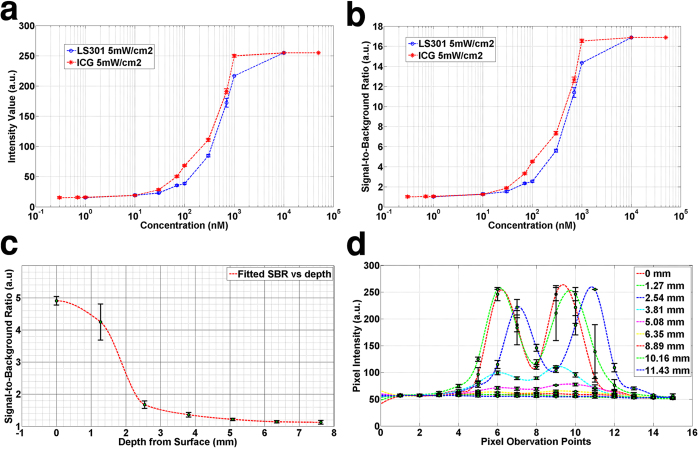
Graphs from phantom experiments for system characterization showing SBR and depth resolution information. (**a**) Fluorescence intensity response with varying concentrations of ICG and LS301. (**b**) The SBR for different concentrations of ICG and LS301. (**c**) SBR for different depths for 1 μM ICG. (**d**) SBR for different depths with 3 mm straws, containing 1μM ICG, positioned 7 mm apart.

We used GAINS to determine the fluorescence intensity profile with increasing concentrations of NIR contrast agents indocyanine green (ICG) and LS301^35^ (Fig. 2a). In the 1 nM to 10 nM range, significant fluorescence was detected, but the signal is close to the sensor noise floor, indicating noise contribution to the net intensity signal. The intensity did not follow a linear trend. A similar profile was observed when signal-to-background ratios (SBRs) were plotted against concentration (Fig. 2b). Therefore, there was no appreciable increase in fluorescence intensity (Fig. 2a) or the SBR (Fig. 2b) from 1 nM to 10 nM. At concentrations higher than 10 nM, we observed a rapid increase in the fluorescence intensity and SBR with increasing molecular probe concentrations. The result suggests that the GAINS was able to detect 1 nM solutions of both ICG and LS301, while maintaining an SBR of ≥1.2 (Fig. 2b). This threshold represents the system’s detection limit in homogenous dimethyl sulfoxide (DMSO) solution.

Analysis of depth and spatial resolution shows that the system is capable of detecting 1 μM ICG inside a tissue mimicking phantom up to a depth of 5 mm and can resolve two 3 mm diameter objects kept 7 mm apart up to a depth of 5 mm with an SBR of ≥1.2 (Fig. 2c,d). At the surface, the SBR for 1 μM ICG was 4.9 (Fig. 2c). This is much lower than the SBR observed for 1 μM solution of ICG in DMSO, which was used to calculate the GAINS’ detection limit (Fig. 2b). Whereas DMSO solvent has minimal scattering and absorption of light at NIR wavelengths, the tissue mimicking liquid phantom (intralipid and India ink^37^) used for depth sensitivity measurement has significantly higher scattering and absorption than DMSO. As a result, the background signals were higher for depth than sensitivity detection experiments, leading to a decrease in the SBR for studies with tissue phantoms at equivalent ICG concentration.

Time needed for complete surgical resection of tumors can vary from minutes to several hours. Surgeons are trained to have very steady head movements during tumor removal, but they may experience involuntary head movement. Such motions could cause inaccurate fluorescence overlay if the image becomes out of focus. Our tests indicate that if an object is within ±2.54 cm of the focal plane of the camera at the typical working distance of 50 cm, the accuracy of the fluorescence overlay on the color image will be within 670 μm. Because involuntary head movement and breathing are within the tested range, the error in fluorescence overlay will be minimal. As a result, there is no need to keep GAINS perfectly stationary during surgery.

### *In vivo* mouse studies

**Figure 3:**
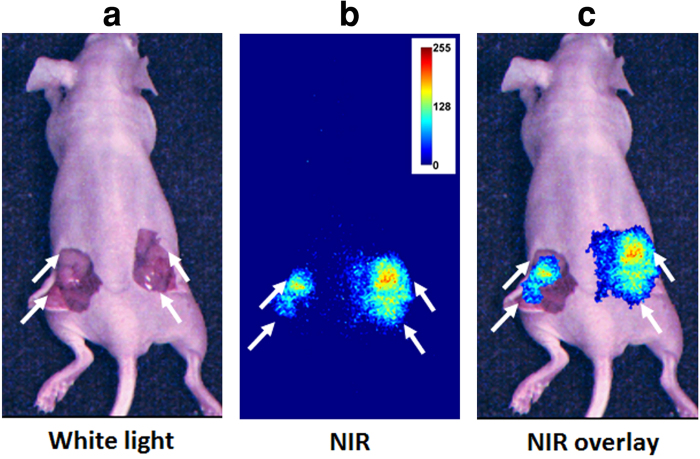
Image-guided tumor visualization in a subcutaneous mouse model. (**a**) Color image of a mouse with skin deflected showing tumor nodes. (**b**) NIR image showing high fluorescence areas. (**c**) Superimposed color-NIR image showing high fluorescence areas accurately correspond to the tumor nodes.

**Figure 4:**
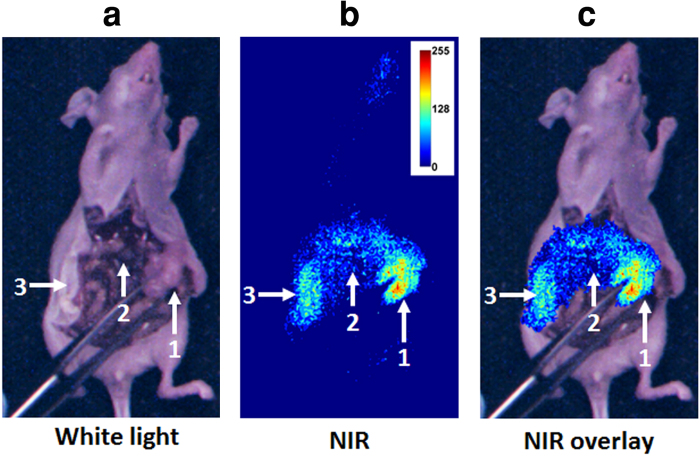
Image-guided exploratory tumor resection in a metastatic mouse model. (**a**) Color image showing a large abdominal tumor (marked 1). (**b**) NIR image showing a high fluorescence area corresponding to the visible tumor (marked 1) and two other areas (marked 2 and 3). (**c**) Superimposed image showing color-NIR overlay image.

**Figure 5:**
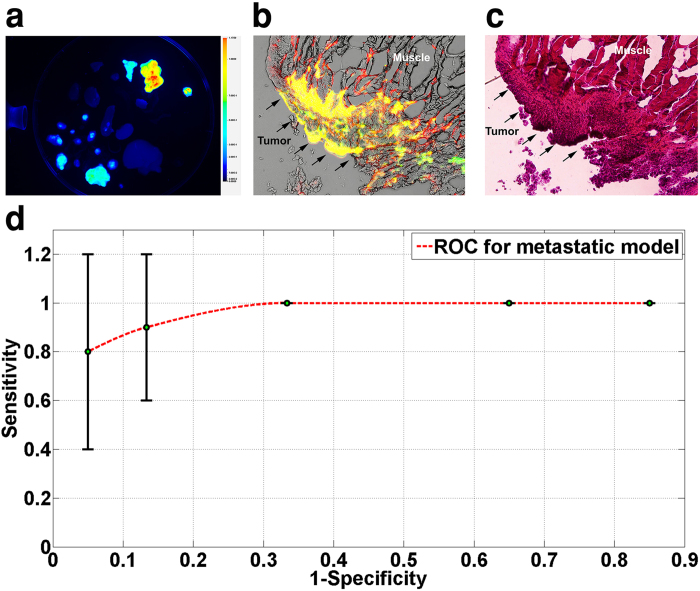
Accuracy of tumor detection in metastatic model. (**a**) iRFP image of harvested organs and tumors from one of the mice showing confirmatory high signal from tumors. (**b**) Fluorescence microscopy revealed good co-localization (yellow) of iRFP signal (green) and LS301 fluorescence (red). (**c**) Histological confirmation of the same slide showing cancerous growth corresponding to the areas marked by iRFP and LS301 fluorescence. (**d**) ROC curve for GAINS tumor detection sensitivity and specificity at different imaging thresholds.

We used a subcutaneous breast cancer mouse model to test *in vivo* GAINS function. Using LS301 fluorescence, the GAINS clearly identified all tumors (n = 10 mice), with a mean SBR of 1.21 ± 0.1 and guided resection in real-time (Fig. 3). The fluorescence signal in the tumors was significantly higher than surrounding tissue (P < 0.05). Histologic analysis confirmed resected tissue as cancerous. In the metastatic mouse model of ovarian cancer, the GAINS identified 27 tumor nodules in 3 mice, with a mean SBR of 1.19 ± 0.03 (Fig. 4), compared to only 10 tumor nodules identified visually alone. Several tumors that were under the visceral organs (3–5 mm deep) were not visible without GAINS guidance. However, real-time visualization of sub-surface fluorescence-guided exploratory surgery revealed tumors that would have been otherwise left behind. The fluorescence signal from suspected tumors were significantly higher than surrounding tissue (P < 0.05), facilitating detection of the smallest tumors (3 mm in diameter). All resected tissues were confirmed to be tumors through infrared fluorescent protein (iRFP)^38^ imaging and histologic analysis, which showed close overlap of iRFP signal with LS301 fluorescence (Fig. 5). Imaging threshold provides guidance in delineating the tumor region for resection (Supplementary Fig. S4) and affects the accuracy of tumor detection. We used receiver operator characteristic (ROC) analysis to determine the best imaging threshold for the metastatic mouse model. The sensitivity and specificity of tumor detection for all images were calculated at several thresholds within the range of 7.8% to 6.3% of maximum pixel intensity. Using the best case threshold for each image, the sensitivity and specificity of tumor detection was calculated to be 100% and 98.33% ± 5%, respectively. Using the average detection sensitivity and specificity for each threshold tested, ROC analysis shows that a threshold of 7.5% of maximum pixel intensity is a reasonable imaging threshold to obtain optimal sensitivity and specificity with GAINS, due to high LS301 uptake in tumor and low tissue autofluorescence (Fig. 5). This threshold was used to identify the tumor region prior to resection. The threshold was manually adjusted during tumor resection to accommodate changes in the residual fluorescence intensities. Our graphical user interface (GUI) has the option of adjusting the threshold of the superimposed images so that only fluorescence intensity above the threshold will be displayed in pseudocolor representation in the color-NIR channel. This approach optimizes the tumor detection sensitivity and specificity for each case.

### Human pilot studies

**Figure 6:**
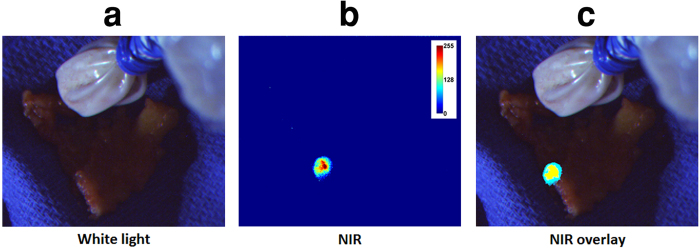
Melanoma patient SLNM showing excised SLN not identified by blue dye. (**a**) Color image showing no blue dye signal although radioactively hot region was detected. (**b**) NIR image showing high fluorescence area. (**c**) Superimposed image showing high fluorescence corresponding to the hot area.

**Figure 7:**
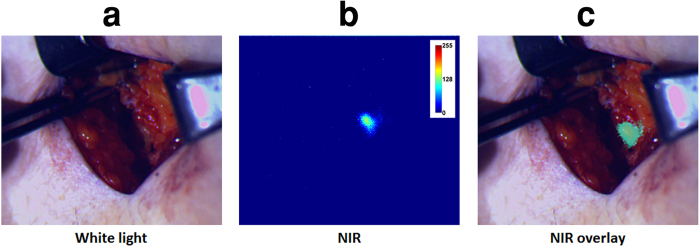
Breast cancer patient SLNM showing non-apparent SLN by visual inspection. (**a**) Color image showing the absence of blue dye. (**b**) NIR image showing high fluorescence area and (**c**) NIR-color superimposed image.

**Figure 8:**
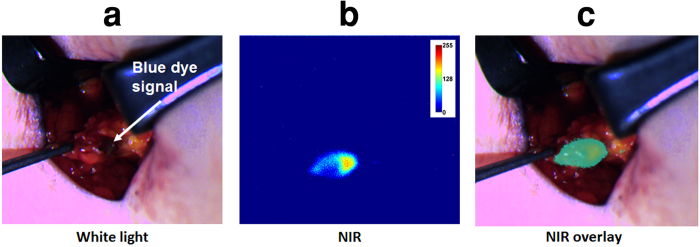
The SLN was apparent after superficial tissue layer was retracted. (**a**) Color image showing retracted tissue layer and visible blue spot from blue dye. (**b**) NIR image showing a larger clear high fluorescence area. (**c**) Color-NIR image showing fluorescence corresponding to the blue dye spot.

Clinical feasibility was demonstrated in 15 patients during SLNM after lumpectomy/mastectomy or wide excision surgeries. Surgeons used the system comfortably, with minimal disruption to the surgical workflow. The GAINS allowed clear visualization of 30 SLNs from 10 breast cancer (Supplementary Vid. S1) and 5 melanoma (Supplementary Vid. S2) patients. Using histologic analysis as the gold standard, the GAINS had a detection sensitivity of 100% in comparison to 92.86% ± 17.5% for the blue dye and 96.43% ± 12.9% for radioactive tracking. There was no statistically significant difference in SLN detection sensitivity by GAINS compared to radioactive tracking (P = 0.34) or blue dye tracking (P = 0.36) methods. In one melanoma patient (Fig. 6), the blue dye did not identify two deep-seated SLNs. Similarly, in one breast cancer patient, initial visual inspection did not reveal the SLN (Fig. 7) and in another patient, radioactive tracking was unable to identify two SLNs. In all these cases, the LNs were clearly identified by GAINS. Although the imaging depth with reasonable resolution is about 5 mm, high fluorescence signal from deep-seated SLNs is readily projected to the surface, allowing visualization of SLNs at >5 mm deep after deflection of the overlying tissue layer (Fig. 8). This finding demonstrates the potential clinical utility of the system for rapid identification of SLNs during surgery.

## Discussion

We have developed a wearable FIGS system that can provide accurate intraoperative visualization of tumors and SLNs in real-time. The ability to detect low NIR fluorescence signal favors the use of GAINS for molecular imaging of low- and high-expression cancer biomarkers. We used lightweight components that are robust, durable and ergonomic. Our compact design allowed dramatic reduction of hardware footprint in the space-starved OR, compared to large standalone systems such as early version FLARE^24^ and SPY^26^ systems. Compact camera design and ergonomic HMD allow wearability and hands-free functionality with minimal training requirements, compared to handheld guidance systems such as Fluobeam^25^ and PDE^27^ that disrupt the normal surgical workflow. The position adjustable camera mounted on the HMD ensures matching of camera and surgeon’s FOV. Our robust image processing algorithm generates composite color-fluorescence images in real-time that are simultaneously displayed on an HMD and an adjacent personal computer (PC) allowing non-disruptive information display to the operating surgeon and simultaneous information availability to the surgical team in the OR. These features are not available in other FIGS systems^24^,^25^,^26^,^27^. The software and GUI are easy to use and compatible with any Windows-based PC. Importantly, superimposed fluorescence information on the normal visual landscape, allows rapid intraoperative visualization of tumors.

In conjunction with LS301, our method clearly identified local and metastatic tumors in murine cancer models, demonstrating the potential for using GAINS to improve the accuracy of tumor resection and decrease the rates of repeat surgeries. Our method allows SLNM with relatively low concentrations of the NIR contrast agent, eliminating exposure to ionizing radiation and minimizing the risk of adverse reactions in patients^9^. The GAINS SLN detection sensitivity was slightly higher than radioactivity and blue dye tracking, although we did not find any statistically significant differences between the methods. Our findings agree with previous studies that have showed ICG fluorescence has comparable or better SLN detection sensitivity compared to radioactivity and blue dye methods^10^,^39^,^40^,^41^,^42^. Although other emerging clinical systems have reported capability of fluorescence detection in the OR, this is the first demonstration of “direct” visualization of NIR fluorescence-color images by surgeons.

A current limitation is the lack of automated focusing, which may lead to image blurring due to large changes in viewing distance. We also currently require a wired connection to a PC for final image processing that restricts the user’s radius of movement. We envision future versions that will automate detection of working distance and adjust the focus according to the working distance of users. We currently use a single camera to capture the user’s FOV, which is displayed in 2D. Future versions will transition to a two-camera stereoscopic system to allow 3D information capture and display for enhanced surgical guidance. We are also developing a robust wireless transmission of image data to enable constraint-free movement. This feature will enable telemedicine applications, remote guidance from experts, and remote training of surgical fellows. Low-cost prototype development and a minimal learning curve for the user favors the use of GAINS in low-resource areas.

In summary, we have developed an ergonomic wearable real-time fluorescence image guidance system that has high detection sensitivity and resolution. The GAINS was able to address successfully the existing limitations of current image guidance systems, including large hardware footprint, field of view mismatch, disruptive information display and real-time image guidance. In conjunction with a tumor-selective NIR probe, the GAINS successfully detected tumors and occult metastatic nodes with high accuracy for guided tumor resection in rodents. Importantly, the GAINS was successfully implemented in the OR for identifying SLNs in human breast cancer and melanoma patients with equivalent or better accuracy than standard methods, although larger sample size is needed to validate this finding. Features such as the non-disruptive real-time image guidance and the need for minimal training will potentially facilitate wide adoption of this technology by clinicians. Further improvements will enable the detection of microscopic lesions in the surgical field, which might otherwise be missed, and possibly prevent damage to nearby uninvolved vital structures such as nerves.

## Materials and Methods

All the animal experiments were conducted in compliance with the requirements for the care and use of laboratory animals in research, and the protocol was approved by the Washington University Animal Studies Committee. The human procedures were carried out in accordance with the approved guidelines by the Institutional Review Board of Washington University. Informed consent was obtained from all patients for this United States Health Insurance Portability and Accountability Act compliant study.

### Contrast Agents

ICG (Cardio green, Sigma-Aldrich, St. Louis, MO) and LS301^35^ were used as NIR contrast agents. Clinical grade ICG for human SLNM was provided by the Siteman Cancer Center (Washington University in St. Louis, MO). ICG and LS301 have similar spectral profiles, allowing the translation of findings with the tumor-targeted LS301 in small animals to humans using FDA- approved ICG, under similar conditions, without major changes in the detection scheme.

### GAINS development

GAINS conceptual design is summarized in Fig. 1a. To maximize spectral separation and minimize light leakage, we recorded an excitation scan of ICG using a fluorimeter (Horiba Jobin) to identify the best excitation wavelengths (Supplementary Fig. S1). We then measured the spectral profiles of LEDs using a spectrometer (Ocean Optics) to identify suitable LEDs and the appropriate excitation and emission filters.

The NIR source (Fig. 1b) consists of 760 nm LEDs (Roithner, Vienna, Austria) with 769 ± 41 nm bandpass filters (Semrock, Rochester, NY). The LED numbers and positions (Supplementary Fig. S2) were optimized using simulations (LightTools). A prototype with a light output of 5 mW/cm^2^ at a distance of 50 cm was used. White flashlights or surgical light (Steris, Mentor, OH) covered with shortpass filters (Cool mirror 330, 3M, St Paul, MN) served as the white light source (Supplementary Fig. S2).

The imaging module collects combined color-NIR signal via a custom F/1.75 glass lens (Supplementary Fig. S3). The incoming signal was divided into visible and NIR components by a custom dichroic beamsplitter cube and directed to a color and NIR complementary metal-oxide semiconductor (CMOS) sensor (Aptina, San Jose, CA). The NIR and color sensors were co-registered (disparity <0.1 mm at 50 cm distance). An 805 nm longpass and a 694 nm shortpass filter (Semrock, Rochester, NY) were placed in front of the NIR and color sensors, respectively, which work in stereoscopic mode (25 MHz clock frequency, 24p frames per second (fps)). A single pair low-voltage differential signaling communicated 16-bits data (8-bits from each sensor) to the processing module at 480 MHz (25 MHz ×18) data transmission rate (Supplementary Table S1).

The processing module consists of a customized printed circuit board connection board and a field-programmable gate array integration module (Opal Kelly, Portland, OR). The printed circuit board powers the imaging module and deserializes the imaging data, which were pre-processed on the field-programmable gate array via optimized Verilog code, and buffered in the on-board 64MB DDR SDRAM. The pre-processed data were transmitted through a high-speed USB 2.0 port to a PC (or laptop). The PC runs C++ program using OpenCV and QT C++ libraries that can execute on any regular Windows x64 PC, without extra software and configuration. The program generates superimposed color-NIR images, creates a GUI that gives access to functions such as display/store/process image data and duplicates images for display on the PC and an HMD module simultaneously. The GUI also allows the use of different exposure times for the photon-saturated visible channel and photon-starved NIR channel, as well as a color correction for best image quality.

The display module consists of a 1080p high-resolution HMD (Carl Zeiss, Oberkochen, Germany). Adjustable mechanical mounting and a counter balance for the imaging module were added to match the camera and user FOV for improved user experience (Fig. 1c).

### *In vitro* phantom studies

All characterization studies were performed at 50 cm imaging distance, 5 mW/cm^2^ illumination, and 24p fps.

For detection sensitivity, spatial average intensity (30 × 30 pixels) was extracted from GAINS images of freshly prepared triplicate samples with different concentrations of ICG (300 pM–50 μM) and LS301 (1 nM–10 μM) dissolved in DMSO and imaged in clear glass vials. The spatially averaged intensity values were plotted against concentration to create the intensity detection profile for GAINS. The SBR was calculated for each concentration by using images acquired in pure DMSO as the background and plotted against dye concentration.

For depth sensitivity, plastic straws of 3 mm diameter were filled with 1 μM ICG and imaged at different depths in a tissue mimicking phantom (μ_a_ = 0.1, 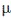
_s_ = 5 cm^−1^), prepared using intralipid and 2% India ink^37^. Pixel intensities corresponding to multiple points on the straw and background were used to calculate SBRs and plotted against depth. For depth resolution, two 3 mm straws filled with 1 μM of ICG were kept 7 mm apart and imaged at various depths in the tissue mimicking phantom. The signal intensities from a cross section of the image were used to create intensity maps and plotted against the depth. Error in color-NIR superimposition was measured by focusing the camera at 50 cm and imaging a target at 50 cm ± 5 cm.

### *In vivo* mouse studies

Six to eight-week-old nude mice (n = 10) were injected subcutaneously into both flanks with 5 × 10^5^ 4T1*luc* murine breast cancer cells. At 5–7 mm tumor size (7–10 days post-implantation), these mice received lateral tail vein LS301 (100 μL, 60 μM in 20% aqueous DMSO) injection. At 1, 4 and 24 h post-injection, the mice were imaged noninvasively using the Pearl small animal imager (LI-COR, Lincoln, NE) and the GAINS. After 24 h, the GAINS was used for intraoperative imaging and image-guided resection of tumors. The resected tissues were preserved for histologic analysis.

Additional 6–8-week-old nude mice (n = 3) were injected intraperitoneally with 1 × 10^7^ SKOV3 human ovarian cancer cells, stably transfected with iRFP^38^. (These cells were kindly provided to us by Dr. Buck E. Rogers, Department of Radiation Oncology at Washington University in St. Louis, MO). When tumors were palpable (5–10 mm, 3 weeks post-implantation), mice received lateral tail vein LS301 (100 μL, 60 μM in 20% aqueous DMSO) injection and were imaged noninvasively using the Pearl system and GAINS at 1, 4, and 24 h post injection. At 24 h post injection, GAINS guided the intraoperative tumor visualization and resection. Resected tissue were imaged for iRFP signal using the Pearl system and then frozen for histologic analysis for determination of GAINS tumor detection sensitivity and specificity. The imaging threshold was varied retrospectively from 6.3% to 7.8% of maximum pixel intensity, and the sensitivity and specificity were calculated for each threshold. ROC analysis was used to calculate the optimal imaging threshold for this model.

### Histology

Fresh-frozen, 10 μm tissue sections were imaged for NIR fluorescence, stained with hematoxylin and eosin (H&E) and the same areas were imaged under brightfield for co-registration with NIR fluorescence using an epifluorescence microscope (BX51 Olympus, Center Valley, PA).

### Pilot human studies

Participants were breast cancer patients (n = 10) undergoing a lumpectomy, partial mastectomy, or radical mastectomy, as well as melanoma patients (n = 5) undergoing wide excision of skin lesions, along with SLNM. Breast cancer patients, were given post-anesthesia, peritumoral injection of a mixture of ^99 m^Tc-sulfur colloid (834 μCi) and methylene blue (5 mL of 1% solution) immediately followed by ICG (5 mg/mL; 5 mL) and site massage for approximately 5 min. At 10–15 min post-injection, the surgeon removed the tumor mass. A handheld gamma probe guided site of axillary incision and invasive SLN identification, which were then examined for the presence of blue color and visualized using the GAINS via ICG fluorescence. The cavity was inspected with the GAINS to identify other fluorescent SLNs, which were then checked for blue color and radioactivity, before excision and preservation for histology. A similar procedure was followed in melanoma patients, except that only 1 mL of ICG solution (5 mg/mL) was injected. In all cases, the GAINS was operated at 24p fps with a 40-millisecond acquisition time. NIR-white light illumination during system usage was provided by our illumination module.

### Statistical Analysis

Statistical analysis was performed using OriginPro8 (OriginLab Corp., Northampton, MA). SBRs, sensitivity, and specificity were expressed as mean and standard deviation. Paired t-tests were used to compare fluorescence signal in tumors and background tissue in mouse models and sensitivity of SLN detection by GAINS, radioactivity, and blue dye methods. P < 0.05 were considered statistically significant.

## Additional Information

**How to cite this article**: Mondal, S. B. *et al.* Binocular Goggle Augmented Imaging and Navigation System provides real-time fluorescence image guidance for tumor resection and sentinel lymph node mapping. *Sci. Rep.* **5**, 12117; doi: 10.1038/srep12117 (2015).

## Supplementary Material

Supplementary Information

Supplementary Video S1

Supplementary Video S2
