# Supplementary material for: Binocular Goggle Augmented Imaging and Navigation System provides real-time fluorescence image guidance for tumor resection and sentinel lymph node mapping
Source: Sci Rep. 2015 Jul 16;5:12117. doi: 10.1038/srep12117 (PMC4503986; doi:10.1038/srep12117)
Supplement: Supplementary Information [file srep12117-s1.docx]

**SUPPLEMENTARY INFORMATION**

**Binocular Goggle Augmented Imaging and Navigation System provides real-time fluorescence image guidance for tumor resection and sentinel lymph node mapping**

Suman Mondal1,2,†, Shengkui Gao3,†, Nan Zhu4†, Gail P. Sudlow1, Kexian Liang1, Avik Som1,2, Walter J. Akers1, Ryan C. Fields5, Julie Margenthaler5, Rongguang Liang4, Viktor Gruev3, & Samuel Achilefu1,2,6,*

1 Department of Radiology, Washington University School of Medicine, St. Louis, MO, USA

2 Department of Biomedical Engineering, Washington University, St. Louis, MO, USA

3 Department of Computer Science and Engineering, Washington University, St. Louis, MO, USA

4 College of Optical Sciences, The University of Arizona, Tuscon, AZ, USA

5 Department of Surgery, Barnes-Jewish Hospital, and the Alvin J. Siteman Cancer Center, Washington University School of Medicine, St. Louis, MO, USA

6 Department of Biochemistry & Molecular Biophysics, Washington University School of

Medicine, St. Louis, MO, USA

^†^These authors contributed equally

^*^Corresponding Author: Samuel Achilefu, PhD, Department of Radiology, 4525 Scott Avenue, Washington University School of Medicine, St. Louis, MO 63110. Phone: 314-362-

8599; Fax: 314-747-5191; E-mail: achilefus@mir.wustl.edu

**Supplementary Table S1.** GAINS specifications

| **Characteristics** | **GAINS Specification** |
| --- | --- |
| Sensor pixel size | 6 µm X 6 µm |
| NIR sensor quantum efficiency | 36% at 810 nm |
| Lens | F/1.75, focal length= 19.6 mm, full diagonal FOV = 15.5° and working distance = 850 mm |
| Disparity | <0.1 mm |
| Weight | 30 gm (camera)  330 gm (camera + HMD) |
| Display | VST HMD |
| Camera | Dual modal complementary metal-oxide semiconductor (CMOS, monochrome & RGB) |
| Processing | FPGA and PC |
| Detection limit | 1 nM ICG, 50 cm, 5 mW/cm^2^ illumination, 24p fps |
| Illumination | 16 LED light, 760 nm, 5 mW/cm^2^ at 50 cm |
| Spatial resolution | 320 µm |
| Depth resolution | 5 mm, 1 µM ICG solution |

**
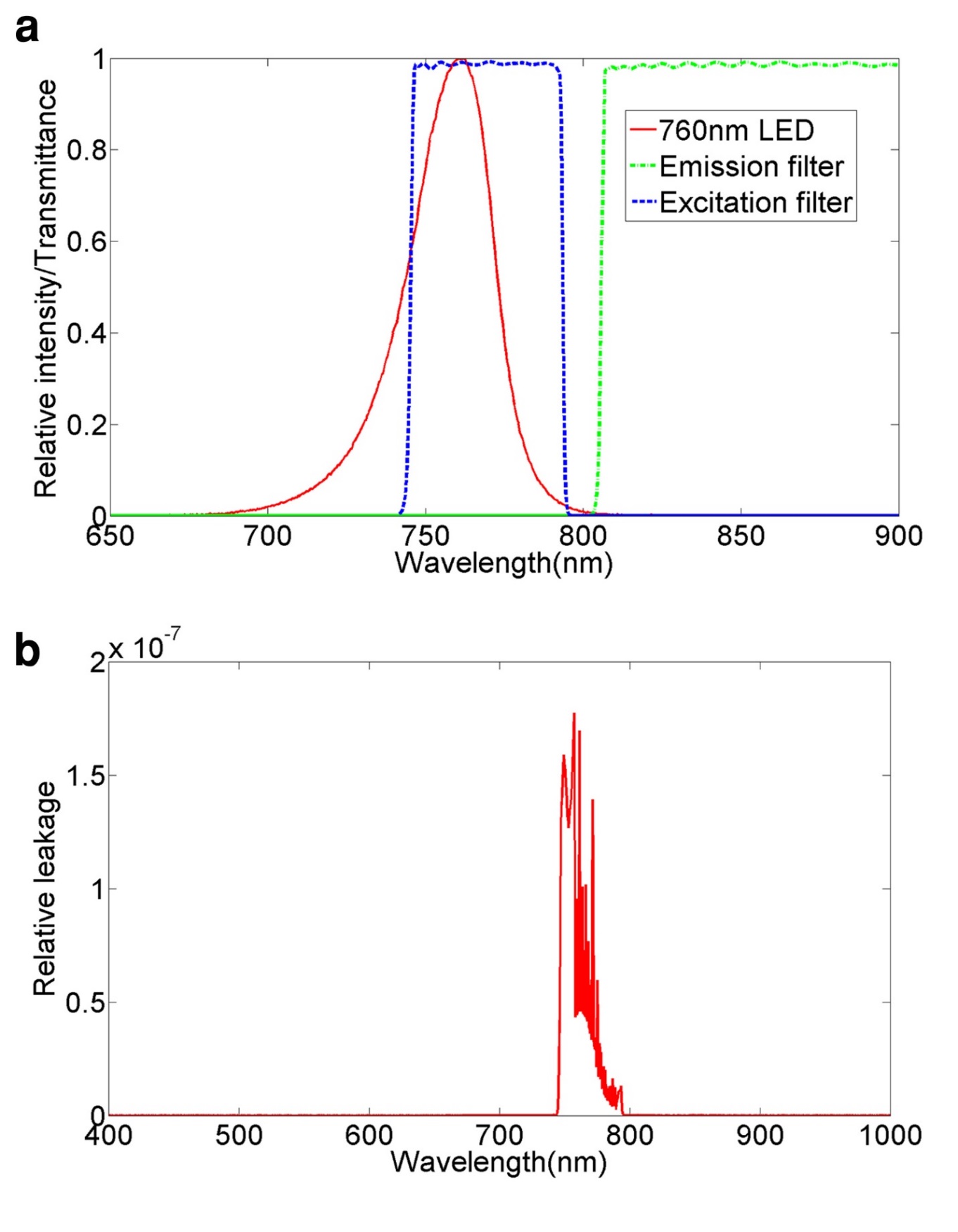
**

**Supplementary Figure S1.** Spectral characterization. (a) Spectral profile of the GAINS system illumination, excitation filter used for illumination and emission filter used for NIR detection showing minimal overlap of the excitation and detected emission spectra. (b) Light leakage relative to NIR LED excitation with our filter choice.


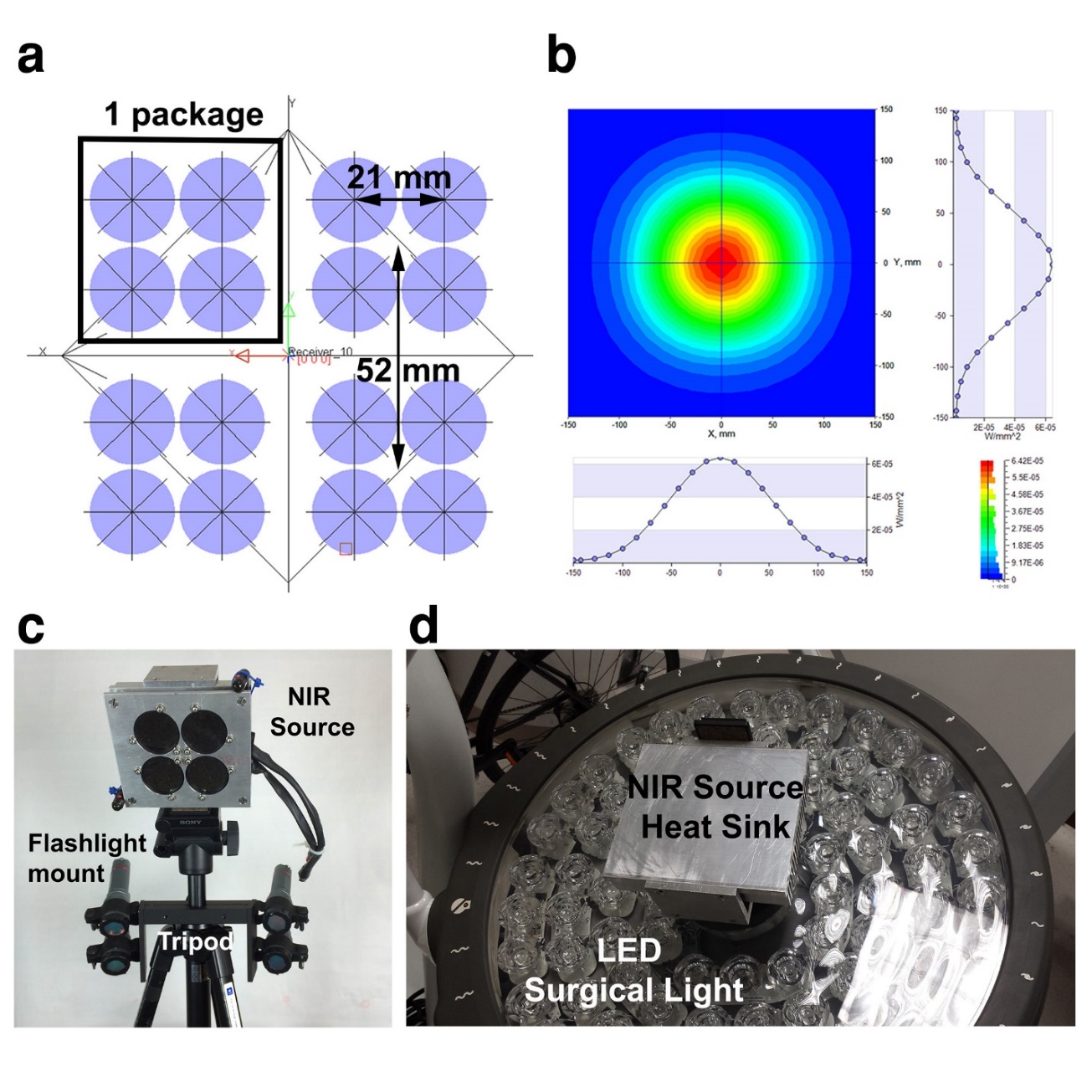


**Supplementary Figure S2.** Illumination module design and prototype. (a) The schematic of the standalone NIR illumination sub-module showing arrangement of 16 LEDs divided in four packages, each consisting of 4 LEDs. Each package has a dimension that allows it to be covered by a 50 mm diameter excitation filter. (b) Simulation result of light output of the four package configuration while running at the typical forward current and at a distance of 50 cm. (c) Tripod configuration with for illumination. Two laser pointers are attached on two corners of the NIR sub-module to easily point the NIR source at the region of interest. A fabricated flashlight mount is used to hold four high power LED flashlights fixed on the central column of the tripod. These flashlights provide the white light illumination for color reflectance imaging. The mount is designed to provide necessary angle for convergence of the flashlight beams with the NIR illumination area at typical working distances. (d) The surgical light configuration uses the threaded back of the NIR light source to attach it to the center of a LED surgical light. The surgical light provides the while illumination for color reflectance imaging. The white LEDs of the surgical light is covered by a filter to cut off the NIR components of the surgical light.


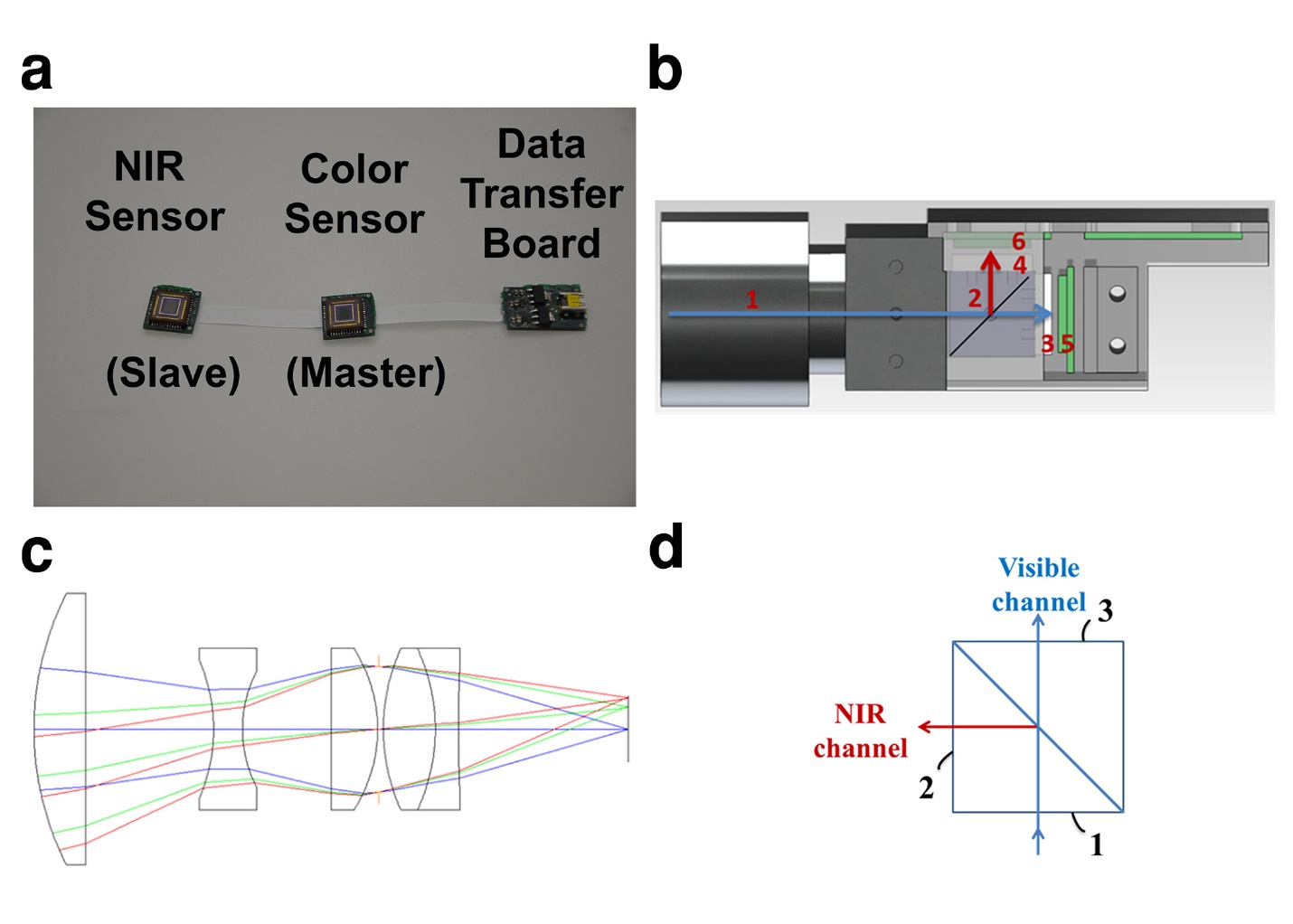


**Supplementary Figure S3.** Imaging module. (a) Imaging module sensors showing the NIR sensor, color sensor and the conversion board. (b) Schematic of Imaging Module internal structure: 1, Lens; 2, dichroic beamsplitter; 3, short-pass filter for visible channel; 4, long-pass filter for NIR channel; 5, visible CMOS sensor; 6, NIR CMOS sensor. (c) Optical structure of the lens. (d) Design specifications of the dichroic beamsplitter: surface 1, broad band AR coating for 450-850 nm; surface 2, near-infrared band (800 nm - 900 nm) AR coating; surface 3, visible band (450 nm - 650 nm) AR coating

**
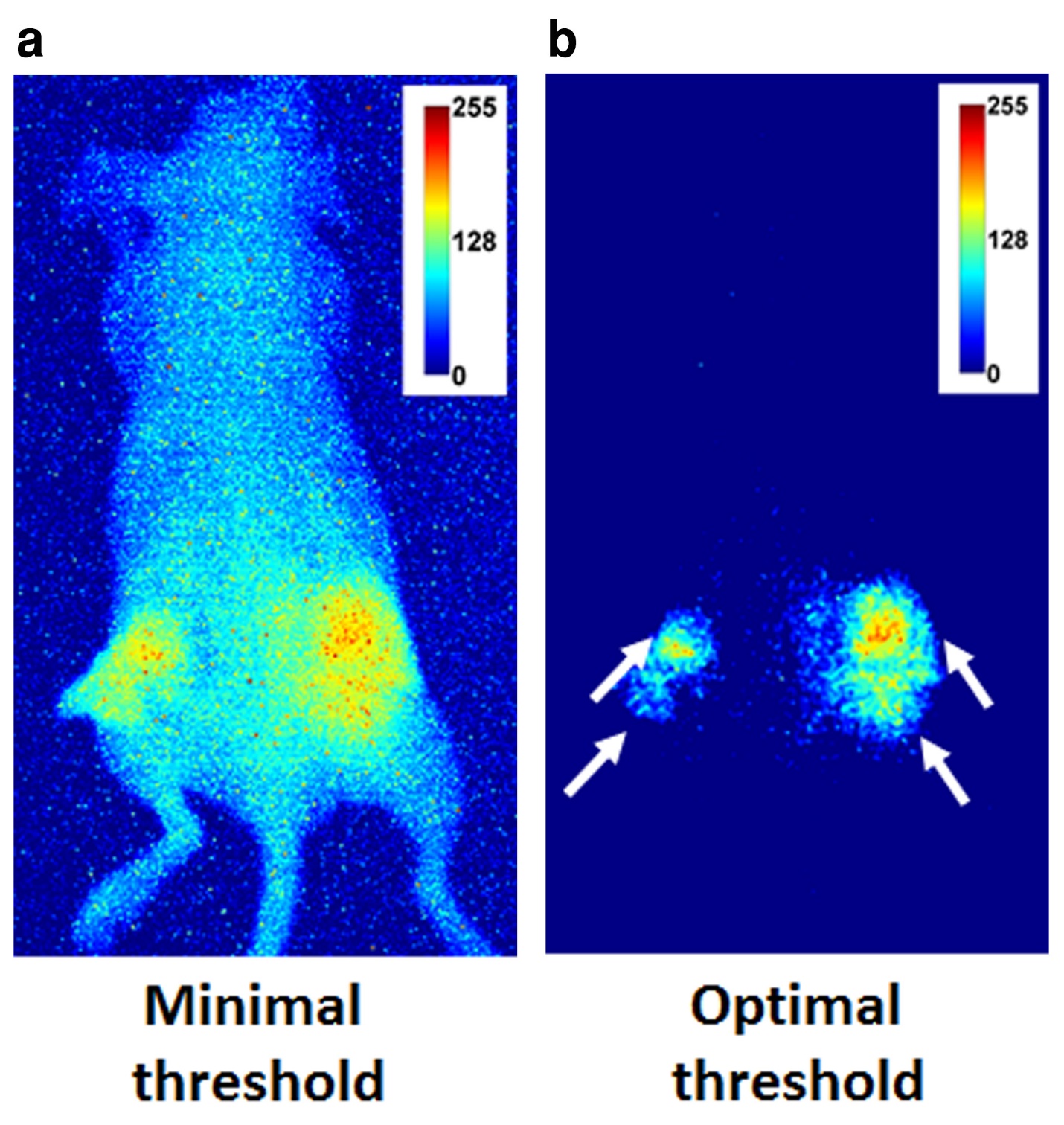
**

**Supplementary Figure S4.** Thresholding during image-guided tumor visualization in subcutaneous mouse model. (a) NIR image with minimal thresholding, showing high fluorescence area corresponding to tumor. (b) NIR image with optimal thresholding, showing well-defined high fluorescence in the tumor region.
